# Comparative physical genome mapping of malaria vectors *Anopheles sinensis* and *Anopheles gambiae*

**DOI:** 10.1186/s12936-017-1888-7

**Published:** 2017-06-05

**Authors:** Yun Wei, Biao Cheng, Guoding Zhu, Danyu Shen, Jiangtao Liang, Cong Wang, Jing Wang, Jianxia Tang, Jun Cao, Igor V. Sharakhov, Ai Xia

**Affiliations:** 1 Department of Entomology, Nanjing Agricultural University, Nanjing, China; 2 Key Laboratory of National Health and Family Planning Commission on Parasitic Disease Control and Prevention, Jiangsu Provincial Key Laboratory on Parasite and Vector Control Technology, Jiangsu Institute of Parasitic Diseases, Wuxi, Jiangsu Province China; 3 Department of Entomology, Fralin Life Science Institute, Virginia Tech, Blacksburg, VA USA; 4 Laboratory for Ecology, Genetics and Environmental Protection, Tomsk State University, Tomsk, Russia

**Keywords:** Chromosomal evolution, GRIMM, Inversion fixation, Fluorescence in situ hybridization, OrthoDB, Synteny blocks

## Abstract

**Background:**

*Anopheles sinensis* is a dominant natural vector of *Plasmodium vivax* in China, Taiwan, Japan, and Korea. Recent genome sequencing of *An. sinensis* provides important insights into the genomic basis of vectorial capacity. However, the lack of a physical genome map with chromosome assignment and orientation of sequencing scaffolds hinders comparative analyses with other genomes to infer evolutionary changes relevant to the vector capacity.

**Results:**

Here, a physical genome map for *An. sinensis* was constructed by assigning 52 scaffolds onto the chromosomes using fluorescence in situ hybridization (FISH). This chromosome-based genome assembly composes approximately 36% of the total *An. sinensis* genome. Comparisons of 3955 orthologous genes between *An. sinensis* and *Anopheles gambiae* identified 361 conserved synteny blocks and 267 inversions fixed between these two lineages. The rate of gene order reshuffling on the X chromosome is approximately 3.2 times higher than that on the autosomes.

**Conclusions:**

The physical map will facilitate detailed genomic analysis of *An. sinensis* and contribute to understanding of the patterns and mechanisms of large-scale genome rearrangements in anopheline mosquitoes.

**Electronic supplementary material:**

The online version of this article (doi:10.1186/s12936-017-1888-7) contains supplementary material, which is available to authorized users.

## Background

*Anopheles sinensis* is a member of the *Anopheles hyrcanus* group within the subfamily *Anophelinae* [1], which has a wide geographical distribution in Asia, mainly including Thailand, Malaysia, Indonesia, Singapore, Vietnam, China, Taiwan, Japan and Korea [2]. Within its range of distribution, *An. sinensis* has been historically considered as the most dominant and important natural vector of *Plasmodium vivax* in China, Taiwan, Japan, and Korea [3]. In China, *An. sinensis* is the most widespread vector of *P. vivax*, with a continuous range throughout 29 provinces and regions [4]. The recent re-emergence of vivax malaria, which started from 2001 in central China, appears in the areas where *An. sinensis* is the only vector [5] and further study reported high susceptibility of *An. sinensis* to *P. vivax* following artificial membrane feeding [6], suggesting that this species is responsible for the recent outbreaks of malaria. After being certified malaria-free, 204,428 and 300,000 cases of malaria in 2000 and 2001, respectively, were found in Korea [3], and three clusters of malaria cases were reported in Singapore in 2009 [7], all of which occurred in regions where *An. sinensis* was the predominant anopheline mosquito. As well as its role in malaria transmission, *An. sinensis* also plays a role in the transmission of a filarial worm, *Brugia malayi*, in China [8, 9].

Advances in next-generation sequencing (NGS) and assembly algorithms have rapidly promoted the analysis of genomes and comparative genomics in anopheline mosquitoes. The whole genome of *An. sinensis* was first published using the Roche/454 GS FLX sequencing approach with a Chinese laboratory strain and assembled into the 9595 scaffolds spanning 220.8 million base pairs (Mb) [10]. At almost the same time, the complete transcriptome of this species was obtained using the Illumina paired-end sequencing technology, and 38,504 unigenes were identified from another Chinese strain [11]. Later, the genome of a different strain of *An. sinensis* (‘SINENSIS’) was sequenced and assembled for comparative analyses by the 16 *Anopheles* mosquito genome project [12]. However, all these research efforts resulted in large numbers of scaffolds and contigs without chromosome assignment or orientations. The availability of a physical map for *An. sinensis* with scaffolds and contigs localized on the chromosomes will increase the quality of comparative genomic analyses with other mosquitoes that have chromosome-based genome assemblies, e.g. *Anopheles gambiae*. Such analyses will allow an exploration of the genomic basis of vectorial capacity and a study of the patterns of chromosome homology and rearrangements between species.

So far, physical maps have been developed for several *Anopheles* mosquito species including *An. gambiae*, *Anopheles funestus*, *Anopheles stephensi*, *Anopheles atroparvus* and *Anopheles albimanus.* These maps improved the draft genome assemblies and helped to understand the genome organization and evolution [13]. *Anopheles gambiae* and *An. funestus* represent two major African malaria vectors, while *An. stephensi* is a dominant vector in Asia. These species belong to the subgenus *Cellia* within the Series, *Pyretophorus* (*An. gambiae*), *Myzomyia* (*An. funestus*), and *Neocellia* (*An. stephensi*) [12]. Comparisons of the mapped genomes of *An. funestus* and *An. stephensi* with the *An. gambiae* genome have demonstrated that the X (sex) chromosome and the 2R arm are much more prone to rearrangement than the other chromosomal arms [14, 15].

Changes in gene order between *An. gambiae* and other species, including *An. atroparvus* and *An. albimanus*, demonstrated that the difference in the rate of evolution between the sex chromosome and autosomes is more than threefold [12]. A recent comparative genomic study between *An. gambiae* within genus *Anopheles* and *Aedes aegypti* in *Culicinae* also revealed that the sex-determining chromosome has a higher rate of genome rearrangements than autosomes [16]. However, whether fast evolution of the sex chromosome occurs in the majority of anophelines will not be clear until more species are investigated.

This study aimed to construct a physical map for *An. sinensis* by anchoring scaffold sequences onto the polytene chromosomes and to identify conserved synteny blocks and fixed inversions between *An. sinensis* and *An. gambiae* for exploring the patterns of chromosome evolution in *Anopheles* mosquitoes.

## Methods

### Mosquito strains and chromosome preparation

The Wuxi laboratory strain (Jiangsu Institute of Parasitic Diseases, Wuxi, China) of *An. sinensis* was used in this study. Polytene chromosome preparations were made using salivary glands dissected from early fourth-instar larvae of *An. sinensis* as previously described [17]. Chromosomes with clear banding patterns were fixed in liquid nitrogen and dehydrated in 50, 70, 90 and 100% ethanol for in situ hybridization.

### Fluorescence in situ hybridization

Genome sequences of the *An. sinensis* China strain were acquired from the database of Zhou et al. [10]. Polymerase chain reaction (PCR) primers for *An. sinensis* scaffolds were designed using the Primer3 Program [18]. PCR procedures were performed with genomic DNA of *Anopheles lesteri* extracted from live fourth-instar larvae with the DNeasy Blood & Tissue Kit (Qiagen GmbH, Hilden, Germany) as templates. After PCR amplification, the PCR products were cut and purified from the agarose gel using a QIAquick Gel Extraction Kit (Qiagen GmbH, Hilden, Germany) and then labelled with either Cy3.5-AP3-dUTP or Cy5.5-AP3-dUTP (GE Healthcare UK Ltd. Chalfont St Giles, UK) using a Random Primed DNA Labelling Kit (Roche Applied Science, Penzberg, Germany). Following the in situ hybridization procedure performed using a previously described method [19], fluorescent signals were detected and recorded with a Zeiss LSM 710 laser scanning microscope (Carl Zeiss Microimaging GmbH, Oberkochen, Germany) and finally mapped to the cytogenetic map of *An. sinensis* [17].

### Gene orthology, syntenic blocks and fixed inversion

OrthoDB was used to identify one-to-one orthologues from *An. sinensis* and *An. gambiae* and to determine their locations on the scaffolds [20]. The comparative positions of the orthologous genes from *An. sinensis* and *An. gambiae* were plotted using genoPlotR [21]. Synteny blocks for each pair of homologous chromosome arms between *An. sinensis* and *An. gambiae* were analysed from the database generated by OrthoDB (Additional file 1). Chromosomal regions containing two or more orthologous genes with the same order and orientations were defined as synteny blocks and numbered 1, 2, 3, etc. along the chromosomes. After obtaining the number of all synteny blocks, the inversion distances on homologous chromosome arms between *An. sinensis* and *An. gambiae* were estimated using the programs of Genome Rearrangements in Mouse and Man (GRIMM) [22].

### Chromosome evolution in *Anopheles* mosquitoes

Chromosome evolution rates represented by inversions/Mb/MY were calculated as inversion number/mapped genome size/divergence time. To compare the evolution rates for each chromosomal arm in different species, previously published data was included for analysis [12]. To explore the fast evolution of sex chromosome, phylogenetic relationships of the 17 anopheline species were considered [12].

## Results

### A physical genome map of *Anopheles sinensis*

**Fig. 1:**
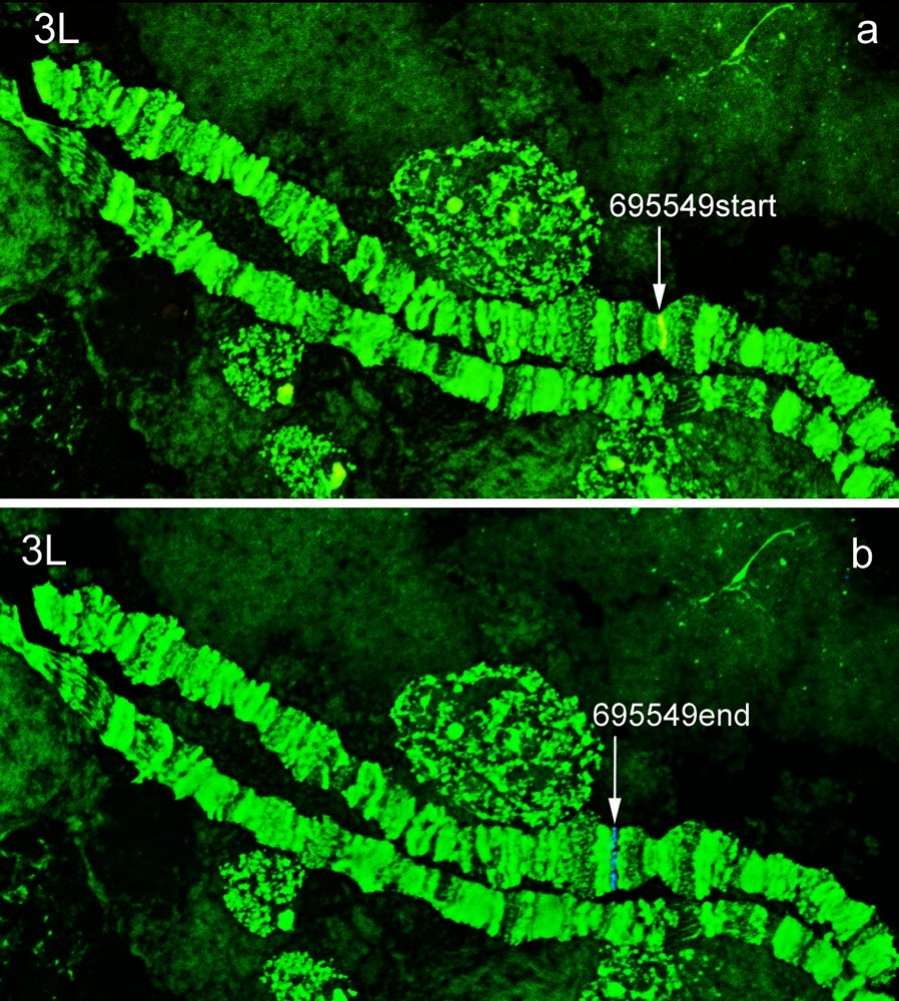
Fluorescence in situ hybridization of the genomic scaffold AS2_scf7180000695549 to the polytene chromosome 3L of *An. sinensis*. Probe 695549start is labelled with a *red* (Cy3.5) dye (**a**) and probe 695549end is labelled with a *blue* (Cy5.5) dye (**b**)

**Fig. 2:**
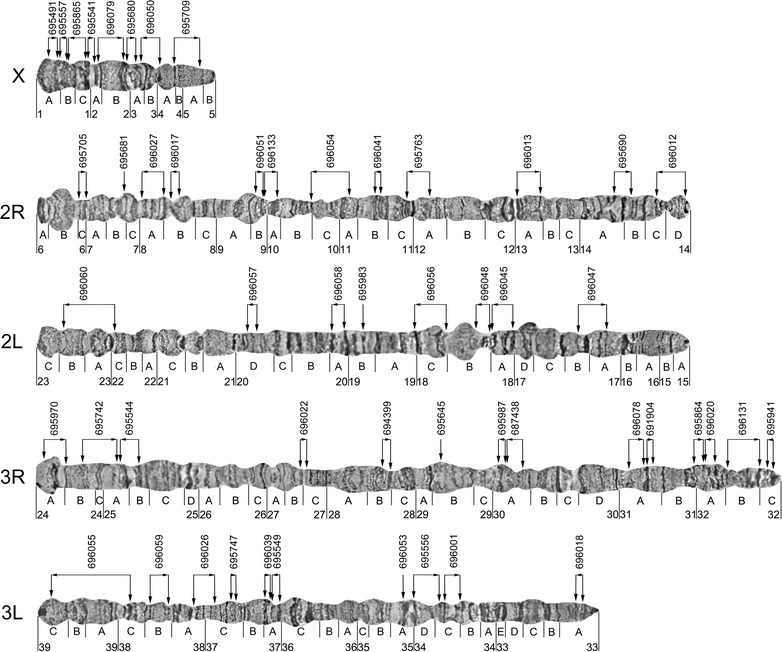
A physical map of the *An. sinensis* genome generated by FISH mapping on polytene chromosomes. *Vertical arrows* indicate the positions of the probes on chromosomes. *Horizontal arrows* show the orientations of scaffolds. The 6-digt numbers are the probe name and the corresponding scaffold name are listed in Table 1. The number and the *A*–*D* at the *bottom* of the map represent the division and subdivisions of the chromosome map

For physical mapping, scaffold sequences were acquired from the database of Zhou et al. [10]. Two pairs of PCR primers were designed from the start and the end of each scaffold. After amplification, the Cy3- and Cy5-labelled probes were hybridized to the polytene chromosomes of *An. sinensis*. Two examples of fluorescence in situ hybridization (FISH), with one clear signal in each, are presented in Fig. 1. A total of 104 clones were mapped to the polytene chromosomes of *An. sinensis* to determine the chromosomal locations of 52 scaffolds. The physical map and scaffold localizations of 52 *An. sinensis* scaffolds are summarized in Fig. 2 and Table 1, respectively. Of the 52 scaffolds, the orientations of 48 scaffolds could be determined, and four scaffolds have unique chromosome locations. This physical map includes 26 of the 30 largest scaffolds. The largest scaffold, AS2_scf7180000696055, with a size of 5,918,260 bp, was mapped to the regions 38C to 39C of the 3L chromosome and the second largest scaffold, AS2_scf7180000696060 (4,138,565 bp) was localized to the 22C-23B of the 2L arm of *An. sinensis* (Table 1). Although X is the shortest chromosome, it had the best mapping coverage among the five chromosomal arms, with eight mapped scaffolds from telomere to centromere, representing 13.42 Mb of genome. Chromosome 2R, 2L, 3R and 3L had 12, 8, 14 and 10 scaffolds, respectively (Table 2). The *An. sinensis* genome physical map composes 79.32 Mb, or 36%, of the total assembled (220.8 Mb) genome sequences (Table 2).

**Table 1:** The localizations of 52 *Anopheles sinensis* scaffolds on the polytene chromosomes

	Clone name on the map	Scaffold name	Chromosome location in *An. sinensis*	Length (bp)
1	695491	AS2_scf7180000695491	X:1A	730,280
2	695557	AS2_scf7180000695557	X:1A_B	1,255,381
3	695865	AS2_scf7180000695865	X:1B_C	2,684,564
4	695541	AS2_scf7180000695541	X:1C_2A	841,721
5	696079	AS2_scf7180000696079	X:2A_B	2,239,752
6	695680	AS2_scf7180000695680	X:2B_3A	1,772,387
7	696050	AS2_scf7180000696050	X:3A_4A	2,422,445
8	695709	AS2_scf7180000695709	X:4A_5A	1,472,510
9	695705	AS2_scf7180000695705	2R:6C	3,132,144
10	695681	AS2_scf7180000695681	2R:7B	3,622,691
11	696027	AS2_scf7180000696027	2R:8A	1,402,797
12	696017	AS2_scf7180000696017	2R:8B	401,984
13	696051	AS2_scf7180000696051	2R:9B	1,646,812
14	696133	AS2_scf7180000696133	2R:9B_10A	2,071,771
15	696054	AS2_scf7180000696054	2R:10C_11A	2,075,225
16	696041	AS2_scf7180000696041	2R:11B	1,460,364
17	695763	AS2_scf7180000695763	2R:11C_12A	1,569,060
18	696013	AS2_scf7180000696013	2R:13A	1,443,628
19	695690	AS2_scf7180000695690	2R:14A_B	1,623,961
20	696012	AS2_scf7180000696012	2R:14C_D	1,036,301
21	696047	AS2_scf7180000696047	2L:17A_B	1,804,022
22	696045	AS2_scf7180000696045	2L:18A	1,433,044
23	696048	AS2_scf7180000696048	2L:18B	1,655,828
24	696056	AS2_scf7180000696056	2L:18C	2,410,210
25	695983	AS2_scf7180000695983	2:19B	1,798,621
26	696058	AS2_scf7180000696058	2L:20A	2,460,545
27	696057	AS2_scf7180000696057	2L:20D	3,056,258
28	696060	AS2_scf7180000696060	2L:22C_23B	4,138,565
29	695970	AS2_scf7180000695970	3R:24A_B	1,982,586
30	695742	AS2_scf7180000695742	3R:24B_25A	1,869,526
31	695544	AS2_scf7180000695544	3R:25A_B	1,280,009
32	696022	AS2_scf7180000696022	3R:27B_C	621,276
33	694399	AS2_scf7180000694399	3R:28B	146,261
34	695645	AS2_scf7180000695645	3R:29B	17,918
35	695987	AS2_scf7180000695987	3R:30A	525,874
36	687438	AS2_scf7180000697438	3R:30A	140,738
37	696078	AS2_scf7180000696078	3R:31A	229,900
38	691904	AS2_scf7180000691904	3R:31A	138,869
39	695864	AS2_scf7180000695864	3R:32A	170,159
40	696020	AS2_scf7180000696020	3R:32A	710,710
41	696131	AS2_scf7180000696131	3R:32B	2,359,367
42	695941	AS2_scf7180000695941	3R:32C	816,509
43	696018	AS2_scf7180000696018	3L:33A	515,011
44	696001	AS2_scf7180000696001	3L:34C	777,379
45	695556	AS2_scf7180000695556	3L:34C_D	1,591,401
46	696053	AS2_scf7180000696053	3L:35A	1,670,191
47	695549	AS2_scf7180000695549	3L:37A	814,231
48	696039	AS2_scf7180000696039	3L:37A	3,601,930
49	695747	AS2_scf7180000695747	3L:37C	566,275
50	696026	AS2_scf7180000696026	3L:37C_38A	1,091,046
51	696059	AS2_scf7180000696059	3L:38B	2,208,365
52	696055	AS2_scf7180000696055	3L:38C_39C	5,918,260

**Table 2:** Genome physical mapping information of *Anopheles sinensis*

Chromosome arm	Scaffolds mapped (n)	Length (bp)	Total sequenced genome (%)
X	8	13,419,040	6.07
2R	12	17,382,848	7.87
2L	8	18,757,093	8.50
3R	14	11,009,702	4.99
3L	10	18,754,089	8.49
Total	52	79,322,772	36.00

The physical map of *An. sinensis* presented in this study was compared with previous mapping data summarized in Table 3. Among mapped anopheline genomes, *An. albiumanus* had the most complete chromosomally anchored genome assembly covering 98.2% of the genome, followed by *An. gambiae*, *An. stephensi* and *An. atroparvus* [12, 23] (Table 3). Mapping of 52 scaffolds in *An. sinensis* and 103 scaffolds in *An. funestus* achieved similar portions of mapped genomes in both species (Table 3). Thus, the new genome map of *An. sinensis* can be used for exploration of chromosomal evolution in malaria mosquitoes.

**Table 3:** Assembly and mapping metrics for anopheline genomes

Species	Genome assembly	Total scaffolds	Mapped scaffolds	Scaffold N50, bp	Total length, Mb	Mapped length, Mb	Mapped, %
*An. albimanus*	AalbS1	204	40	18,068,499	170.5	167.4	98.2
*An. gambiae*	AgamP4	8	5	49,364,325	264	273.1	84.3
*An. stephensi*	AsteI2	23,371	86	1,591,355	221.3	137.14	62
*An. stephensi*	AsteS1	1100	101	837,295	225	92.83	41
*An. atroparvus*	AatrE1	1371	7	9,206,694	224.3	88.8	39.6
*An. sinensis*	AsinC2	9592	52	814,231	220.8	79.3	35.9
*An. funestus*	AfunF1	1392	103	671,960	225.2	79.0	35.1

Data for *An. sinensis* are from this study. Data for *An. albimanus* and *An. stephensi* AsteI2 are from Ref. [26] and Ref. [23], respectively. Data for *An. gambiae* are from Ref. [25] and https://www.vectorbase.org/organisms/anopheles-gambiae/pest/agamp4. Data for other species are from Ref. [12]

### Synteny and gene order evolution in *An. sinensis* and *An. gambiae*

**Fig. 3:**
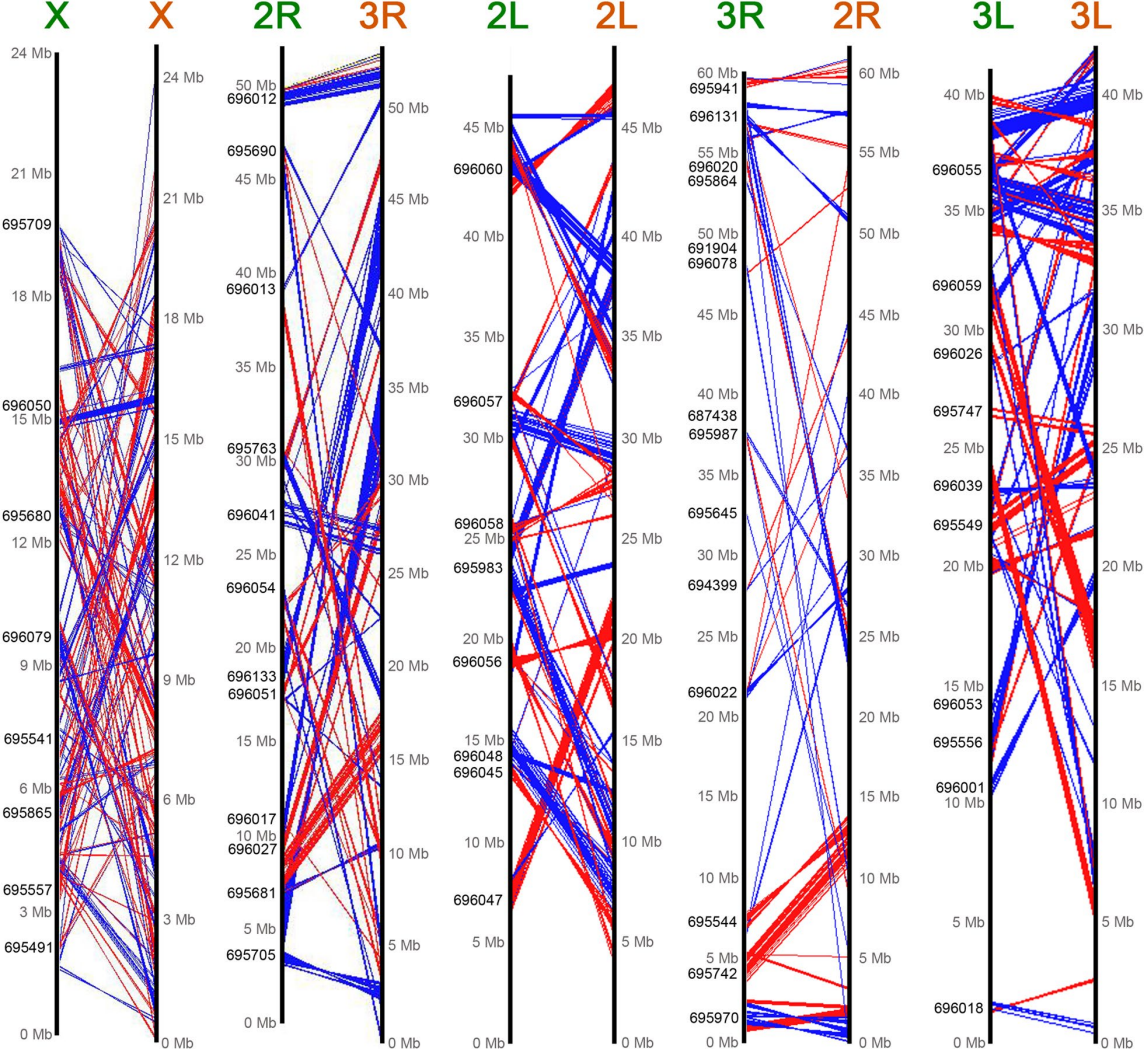
The relationship of the positions of orthologous genes between the *An. sinensis* and *An. gambiae plotted* with GenoPlotR. Orthologues with the same orientation in *An. sinensis* (*left side*) and *An. gambiae* (*right side*) are connected with *red lines*, and orthologues with the opposite orientation are connected with *blue lines*

A total of 3955 one-to-one orthologues were identified from *An. sinensis* and *An. gambiae* using OrthoDB [20] (Additional file 1). The comparative positions of genes within mapped scaffolds based on orthology relationships were plotted on *An. sinensis* and *An. gambiae* chromosomes using genoPlotR [21] (Fig. 3). Physical mapping data were used to determine the orientations of scaffolds, and the default orientations were assigned to some scaffolds with only one probe. Figure 3 shows that the gene orders were reshuffled on five chromosome arms because of fixed inversions. The gene order changes on the X chromosome were more dramatic than those on the autosomes: 2R, 2L, 3R and 3L. The comparative chromosomal locations and orientations of 3955 orthologous genes were further used to determine the number of synteny blocks in the two species. Synteny blocks were defined as genomic regions containing at least two orthologous genes with the same order and orientation. A total of 364 synteny blocks have been identified between *An. sinensis* and *An. gambiae* (Table 4). The analysis revealed that the average length of 112 synteny blocks on the X chromosome (85,989 bp) is much smaller than those on the remaining chromosomes (237, 175; 239, 627; 197, 751 and 242, 299). Additionally, the largest synteny block on the X arm is only 766,489 bp, whereas the largest block on 2R is 1,796,395 bp, which is twice that on the X arm (Table 4). These results suggest that the sex X chromosome has smaller synteny blocks than the autosomes.

**Table 4:** The synteny blocks between *An. sinensis* and *An. gambiae* using *An. sinensis* as reference

Chromosome	Number of synteny blocks (n)	The average length of synteny blocks (bp)	The largest synteny blocks (bp)	The smallest synteny blocks (bp)
X	112	85,989	766,489	1676
2R	68	237,175	1,796,395	1796
2L	70	239,627	1,629,455	1514
3R	47	197,751	1,709,848	3361
3L	67	242,299	1,469,290	4748
Total	364	191,358	1,474,295	2619

To further analyse fixed inversions between *An. sinensis* and *An. gambiae*, we input the order of 361 synteny blocks (Additional file 2) into the Genome Rearrangements in Man and Mouse (GRIMM) program [22]. Table 5 shows that a minimum of 267 inversions were estimated between *An. sinensis* and *An. gambiae*. The sex chromosome exhibited a greater number of inversions (101), whereas the autosomes 2R, 2L, 3R and 3L had 42, 51, 33 and 40 inversions, respectively (Table 5). The total size of mapped scaffolds on each *An. sinensis* chromosome was used to calculate the density of inversions per megabase. Our data demonstrate that the inversion breaks per megabase on X chromosome is 7.527, which is approximately 3.2 times greater than the average density of inversions on autosomes (2.367) (Table 5). Among the autosomes, the inversion density between *An. sinensis* 3R and *An. gambiae* 2R is 2.997 inversions/Mb, which is higher than for the remaining autosomes. The 3L chromosome exhibits the lowest density of inversions (2.133 inversions/Mb). The most recent study of the chromosome evolution in *Anopheles* used the divergence time between *An. atroparvus* and *An. gambiae* of 58 MY [12], and *An. atroparvus* and *An. sinensis* belong to the subgenus *Anopheles*. The divergence time of 58 MY was used to calculate the number of inversions per magabase per million years (inversions/Mb/MY) (Table 5). For *An. sinensis* and *An. gambiae*, the rates of evolution were 0.130 for X and 0.042, 0.047, 0.052, 0.037 for 2R, 2L, 3R and 3L, respectively (Table 5).

**Table 5:** Fixed inversions between *An. sinensis* and *An. gambiae*

Chromosome name in *An. sinensis*	Size of mapped scaffolds in *An. sinensis* (Mb)	Inversions (GRIMM)	Inversions/Mb	Inversions/Mb/MY (divergence time 58 MY)
X	13.419	101	7.527	0.130
2R	17.382	42	2.416	0.042
2L	18.757	51	2.719	0.047
3R	11.010	33	2.997	0.052
3L	18.754	40	2.133	0.037
Total	79.322	267	3.366	0.058

### Rapid evolution of the sex chromosome in *Anopheles* mosquitoes

**Fig. 4:**
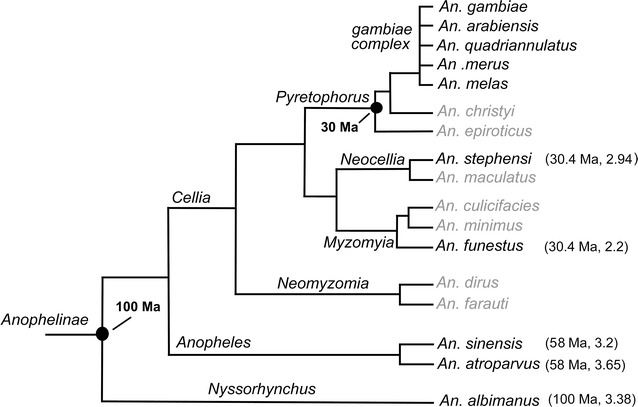
Reconstructed phylogenetic relationships of the 17 anopheline species and chromosomal evolution analysis from Ref. [12]. The aligned protein sequences of 1085 single-copy orthologs were used to construct the maximum likelihood molecular phylogeny. Chromosome evolution analysis was conducted between the species indicated with a *dark font* and *An. gambiae*. Comparative physical mapping has not been performed for the species marked with a *grey font*. *Ma* represents million years ago. The* number in brackets* after the divergence time is the ratio of the X chromosome evolution rate to the autosomal rate of rearrangements in each species compared with *An. gambiae*

To understand the pattern of inversion fixations in malaria mosquitoes, the number of inversions/Mb/MY in our analysis was compared with the earlier published data [12] (Table 6). The results revealed that inversion rates on autosomes varied between *An. gambiae* and each of five *Anopheles* species. However, the density of fixed inversions on the X was consistently greater than that on autosomes (Table 6), suggesting the faster evolution of X chromosome in *Anopheles* mosquitoes. The ratio of the X chromosome evolution rate to the autosomal rate of rearrangements in *An. sinensis* and *An. gambiae* was also calculated and our data demonstrated that the X chromosome evolved approximately 3.2 times faster than autosomes. Our chromosomal evolution analysis data was added into the phylogenetic relationships of the 17 anopheline species constructed by Neafsey et al. [12] using the aligned protein sequences of 1085 single-copy orthologs. Figure 4 shows that the ratio of the X chromosome evolution to the autosomal rate of rearrangements varies among the *Anopheles* lineages with being higher in subgenera *Anopheles* and *Nyssorhynchus* and lower in genus *Cellia*.

**Table 6:** The rates of inversion fixation between *An. gambiae* and other *Anopheles* species

Species	X	2R	2L	3R	3L
*An. albimanus*–*An. gambiae*	0.130	0.043	0.040	0.034	0.037
*An. atroparvus*–*An. gambiae*	0.124	0.036	0.035	0.025	0.041
*An. funestus*–*An. gambiae*	0.121	0.064	0.065	0.026	0.066
*An. sinensis*–*An. gambiae*	0.130	0.052	0.047	0.042	0.037
*An. stephensi*–*An. gambiae*	0.128	0.049	0.036	0.028	0.036

The rates represent the number of inversions per Mb per MY. Data for *An. sinensis* are from this study. Data for other species are from Ref. [12]

## Discussion

### A physical map is a critical tool for improving a genome assembly and for studying chromosomal evolution

In this study, a physical map was constructed for an Asian malaria vector *An. sinensis* using fluorescence in situ hybridization (FISH) of DNA probes with polytene chromosomes. The physical mapping of *An. sinensis* placed 52 large scaffolds with total length of 79,322,722 bp from the genome database to the chromosomes (Fig. 2; Table 2). It accounted for approximately 36% of the total assembled (220.8 Mb) genome sequences of *An. sinensis* (Table 2).

So far, several genome maps have been developed for malaria mosquitoes and we compared the percentage of the physically mapped genome in *An. sinensis* with data from other species [12, 23]. Among mosquitoes, the African malaria vector *An. gambiae* was the first to have its genome sequenced [24]. More than 2000 BAC clones were originally placed onto the chromosomes for genome mapping and later, additional mapping added small scaffolds to the area around the centromeres, which resulted in ~84.3% of the *An. gambiae* genome assembly [25]. The physical map of *An. albimanus* initially placed ~76% of genome onto the chromosomes [12], while a more recent physical mapping effort reached the 98.2% coverage of the *An. albimanus* genome assembly [26], which is the most complete genome assembly to date. The genome of *An. stephensi*, a key vector of malaria throughout the Indian subcontinent and Middle East, has also been sequenced and assembled. A total of 86 scaffolds were in situ hybridized to the polytene chromosomes of *An. stephensi*, representing 62% of the genome assembly [23]. *Anopheles atroparvus* and *An. funestus* had mapped portions covering 39.6 and 35.1% of the total genome, respectively [12]. In this research, our new physical map for *An. sinensis* covers 35.9% of the genome, which is within the range of other *Anopheles* species (Table 3).

### Fast evolution of the sex chromosome in *Anopheles* mosquitoes

The availability of the genome sequences and physical maps for *Anopheles* mosquitoes have promoted detailed analysis of the patterns of fixed inversions [12, 13]. In our study, 361 conserved synteny blocks and 267 fixed inversions were identified between *An. sinensis* and *An. gambiae*. Analysis of the density of inversions per Mb and the rate of chromosomal rearrangements in *An. sinensis* and *An. gambiae* suggested that fast evolution occurs on the sex chromosome. The earliest study of inversions on closely related species of the *An. gambiae* complex revealed that 5 of 10 inversions were on the X chromosome, providing the first evidence of fast evolution of sex chromosomes in *Anopheles* mosquitoes [27]. Several species belonging to different series within the subgenus *Cellia* have been extensively studied: *An*. *gambiae* (*Pyretophorus*), *An. stephensi* (*Neocellia*) and *An. funestus* (*Myzomyia*) [12]. The comparative analysis between *An. funestus* and *An. gambiae* as well as between *An. stephensi* and *An. gambiae* [14, 15] further demonstrated that the X chromosome evolved faster than the autosomes. The most recent analyses based on the genome assembly confirmed that the rate of evolution on X is approximately 2.2 times faster than the average autosomal rate for *An. funestus* and *An. gambiae* [12] or 2.94 times faster for *An. stephensi* and *An. gambiae* [23] (Fig. 4). *Anopheles sinensis* and *An. atroparvus* are members of the subgenus *Anopheles*, which is thought to have diverged from *An. gambiae* 58 MY ago [10, 12]. Previous studies have shown that the difference in the rate of evolution between the sex chromosome and autosomes is approximately 3.65 times in *An. atroparvus* and *An. gambiae* [12]. In this study, the density of inversions on the X chromosome is found to be 3.2 times greater than the average density of inversions on the autosomes between *An. sinensis* and *An. gambiae* (Fig. 4). These results suggest that the rapid evolution of sex chromosome is a common feature in *Anopheles* mosquitoes. The X chromosome rearrangements may play a role in speciation of malaria mosquitoes [14, 28]. Future genome studies can provide valuable information for dissecting the role of X chromosome inversions in speciation of malaria vectors.

## Conclusions

This study constructed a physical genome map for an important malaria vector of *P. vivax*, *An. sinensis*, which is the most widely distributed vector in China, Korea, and Japan. This physical map includes 52 of the largest scaffolds from *An. sinensis*, spanning approximately 80 Mb of the 220 Mb, or approximately 36%, of the sequenced genome. The map coverage is similar to the mapped portion of *An. funestus* and *An. atroparvus*. By analysing the comparative positions of 3955 orthologous genes, 361 conserved synteny blocks and 267 fixed inversions between *An. sinensis* and *An. gambiae* were identified. The rate of evolution of the sex chromosome is approximately 3.2 times greater than the average autosomal rate of evolution. Thus, our comparative analysis in *An. sinensis* and *An. gambiae* inferred from physically mapped genome assemblies provided additional details for understanding chromosome evolution in malaria vectors.

## Additional files

**Additional file 1.** Orthologous genes in *An. sinensis* and *An. gambiae*.

**Additional file 2.** The orders of synteny blocks for running GRIMM.
