# Supplementary material for: Comparative physical genome mapping of malaria vectors Anopheles sinensis and Anopheles gambiae
Source: Malar J. 2017 Jun 5;16:235. doi: 10.1186/s12936-017-1888-7 (PMC5460330; doi:10.1186/s12936-017-1888-7)
Supplement: Supplementary file 2 — Additional file 2. The orders of synteny blocks for running GRIMM. [file 12936_2017_1888_MOESM2_ESM.docx]

**Additional file 2: The orders of synteny blocks for running GRIMM**

***An. sinensis* X**: 1 2 3 4 5 6 7 8 9 10 11 12 13 14 15 16 17 18 19 20 21 22 23 24 25 26 27 28 29 30 31 32 33 34 35 36 37 38 39 40 41 42 43 44 45 46 47 48 49 50 51 52 53 54 55 56 57 58 59 60 61 62 63 64 65 66 67 68 69 70 71 72 73 74 75 76 77 78 79 80 81 82 83 84 85 86 87 88 89 90 91 92 93 94 95 96 97 98 99 100 101 102 103 104 105 106 107 108 109 110 111 112

***An. gambiae* X**: -10 -76 -1 -15 -57 -56 17 18 86 -53 16 -93 -92 -101 74 -24 50 23 -95 108 105 -107 -58 -14 59 -42 -69 109 -20 -35 71 19 -21 54 48 -85 98 -44 -43 -5 6 -34 37 104 29 -33 75 64 62 72 81 -79 61 -102 -91 90 -89 -12 -87 -51 -112 -36 -2 -97 -25 -67 -66 -65 -7 -78 11 100 -70 -94 38 -103 31 26 -40 3 -32 -111 -13 -28 -9 -52 47 -45 99 88 -46 39 41 -110 49 63 96 -30 -106 -22 -8 83 -84 -55 -60 68 -82 -80 -77 -73 -27 -4

112 genes      Reversal Distance: 101

***An. sinensis* 2R**: 1 2 3 4 5 6 7 8 9 10 11 12 13 14 15 16 17 18 19 20 21 22 23 24 25 26 27 28 29 30 31 32 33 34 35 36 37 38 39 40 41 42 43 44 45 46 47 48 49 50 51 52 53 54 55 56 57 58 59 60 61 62 63 64 65 66 67 68

***An. gambiae* 3R**: 41 -36 -2 -1 -3 -40 54 -25 -61 -60 -59 -58 -57 29 -37 52 53 -39 -16 14 15 -13 64 65 -28 45 19 50 43 -26 -51 18 42 20 -17 -38 30 23 24 62 -12 11 -8 9 -10 -7 -6 -5 -4 -63 27 -35 34 -33 -32 22 44 21 -31 -49 -48 47 -46 -56 -55 66 -67 -68

68 genes      Reversal Distance: 42

***An. sinensis* 2L**: 1 2 3 4 5 6 7 8 9 10 11 12 13 14 15 16 17 18 19 20 21 22 23 24 25 26 27 28 29 30 31 32 33 34 35 36 37 38 39 40 41 42 43 44 45 46 47 48 49 50 51 52 53 54 55 56 57 58 59 60 61 62 63 64 65 66 67 68 69 70

***An. gambiae* 2L**: 53 31 64 -60 -34 33 -32 -63 54 49 -41 -47 30 -56 -62 55 -57 -48 65 26 -27 28 21 -25 -70 58 -69 -68 -67 -66 -52 51 -50 40 17 3 -38 39 19 61 -24 -23 -4 5 -6 7 -8 9 -2 -18 -46 45 -44 43 -42 -10 -29 -20 -59 22 -37 36 -35 -1 -11 12 -13 14 15 16

70 genes      Reversal Distance: 51

***An. sinensis* 3R**: 1 2 3 4 5 6 7 8 9 10 11 12 13 14 15 16 17 18 19 20 21 22 23 24 25 26 27 28 29 30 31 32 33 34 35 36 37 38 39 40 41 42 43 44 45 46 47

***An. gambiae* 2R**: -38 -4 -3 -2 -8 -7 -6 -5 1 9 12 11 27 -28 -26 -31 10 -15 -40 -44 -37 36 35 -34 29 30 -22 -21 -20 13 -16 41 -25 -33 23 14 24 -17 -43 32 18 19 42 -45 -39 -47 -46

47 genes      Reversal Distance: 33

***An. sinensis* 3L**: 1 2 3 4 5 6 7 8 9 10 11 12 13 14 15 16 17 18 19 20 21 22 23 24 25 26 27 28 29 30 31 32 33 34 35 36 37 38 39 40 41 42 43 44 45 46 47 48 49 50 51 52 53 54 55 56 57 58 59 60 61 62 63 64 65 66 67

***An. gambiae* 3L**: -66 67 -39 -38 -5 26 33 48 49 40 -27 -34 -35 -32 -29 -28 -51 52 65 53 -50 41 -7 62 -61 -46 -45 -37 54 55 56 57 58 59 60 47 36 -64 23 3 24 -18 17 -16 -15 6 63 -22 9 10 21 -8 31 30 42 1 -43 25 -4 -2 44 -14 19 -11 -20 12 -13

67 genes      Reversal Distance: 40
